# Perceptions of mothers on child well-being, changes in everyday life and social sustainability: lessons learned from a community-based health promotion programme in Anuradhapura District, Sri Lanka

**DOI:** 10.1186/s41043-022-00295-w

**Published:** 2022-05-13

**Authors:** Najith Duminda Galmangoda Guruge, Adam Arhelger, Kalpani Abhayasinghe

**Affiliations:** 1 Department of Health Promotion, Faculty of Applied Sciences, Rajarata University of Sri Lanka, Anuradhapura, Sri Lanka; 2 Public Health and Health Economics, Göteborg, Sweden; 3 Department of Nursing and Midwifery, General Sir John Kotelawala Defence University, Ratmalana, Sri Lanka

**Keywords:** Health promotion, Health literacy, Child well-being, Social sustainability

## Abstract

**Background:**

From 2012 to 2015, a health promotion intervention (under a project called HADP) aiming to improve children’s well-being was implemented in Horowpathana, Sri Lanka. The donor organization reports positive results regarding children’s well-being and behaviour changes, but mixed results regarding its sustainability. A need for a complementary evaluation was therefore identified.

The current study intended to provide a complementary evaluation at four months after the programme closure and to assess the sustainability of the HP intervention from the perspective of mothers who participated in the HADP. Ethical approval for this study was obtained from the ethics review subcommittee of Faculty of Applied Sciences, RUSL.

**Methods:**

A descriptive qualitative study was carried out using in-depth, semi-structured interviews with a convenient sample of 15 mothers who previously participated in HADP. Data analysis was done using the content analysis method.

**Results:**

Mothers attributed diverse perceptions in line with the theme of “health literacy”. Two sub-themes emerged: transformation for betterment and sustainability. The sub-theme of transformation for betterment consists of three categories: individual-level transformation, family-level transformation and social/community-level transformation. Sub-theme sustainability consists of two categories: (1) drivers/adaptations for continuation and (2) determinants that hinder the continuation.

**Conclusions:**

The mothers’ perspectives were strongly related to the definition of health literacy, which emphasizes people’s ability to use health information to make “well-informed” decisions that incorporate a public health perspective. They also acknowledged the responsibility of social organizations to address health literacy. From a health promotion perspective, the findings of our study indicate that people and organizations can use their health literacy skills to improve the health and well-being of their community and its members. Further research is necessary to explore the factors that affect the sustainability of health promotion interventions in the long run.

**Supplementary Information:**

The online version contains supplementary material available at 10.1186/s41043-022-00295-w.

## Background

Sri Lanka can be considered as a model for economically challenged countries that has a remarkable development of the health sector [1]. Citizens of Sri Lanka have access to free universal health care. Innovative and evidence-based approaches are used in health care settings [1]. The National Strategic Framework for Public Health is in line with the Sustainable Development Goals (SDG) [2, 3]. Both government and non-government bodies carry out health promotion programmes for children, women and lay communities in rural and deprived settings [4–6].

Sustainable development is commonly described as containing three reciprocal factors: economic, social and environmental [7, 8]. Health promotion has a strong focus on social sustainability which refers to “a system of social organization that alleviates poverty” ([7], p. 152). Sustainability is defined as the implementation of health-related interventions: “(1) after a defined period of time, (2) the program, clinical intervention, and/or implementation strategies continue to be delivered and/or (3) individual behaviour change (i.e. clinician, patient) is maintained; (4) the program and individual behaviour change may evolve or adapt while (5) continuing to produce benefits for individuals/systems” ([9], p. 5).

The concept of sustainable development has been operationalized in the framework of Dahlgren and Whitehead [9], addressing the complexity of social, economic and environmental factors. The Ottawa Charter defines health promotion as “the process of enabling people to increase control over *the determinants of health* and thereby improve their health” [10]. Health promotion addresses social determinants of health such as poverty, sexism [10] and promoting the empowerment of people [11]. According to the WHO, empowerment in health promotion is crucial [10] as it addresses power relations and social change [12] and links individual well-being with the socio-political context of a country [13, 14]. Community-based approaches are key components of health promotion [10, 15, 16]. Ideally, the community of concern identifies issues, controls the process and is technically supported by professionals who evaluate it [17, 18]. The community “owns” the intervention [16].

Well-being contains subjective and objective indicators, and it can measure diverse things as happiness, health and prosperity which are contextually dependent [19–23]. In order to evaluate well-being, one should consider the broader context as otherwise, global inequalities may be blurred [19], de-politicized and thus worsen the situation [20]. Subjective well-being complements objective measures by offering the “dimension of how individuals feel about their health or economic status” (p. 3) [20]. Well-being in general highlights the determinants of health within health promotion [21, 24] including the community’s role [20].

### Horowpathana area development programme (HADP) by World Vision Sri Lanka (WVSL)

Horowpathana is a divisional secretariat division (DSD) located in Anuradhapura district, which consist of 100 villages. The total population of Horowpathana DSD is 42,169 people. There are about 8000 families (25, 767 individuals) living in the programme impact area. About 25% of the population live below or close to the official district poverty line (a proxy indicator: reception of government assistance) [4]. Twenty-two per cent of the population speak Tamil language, while 88% speak Sinhala [22]. Paddy cultivation and wage-based employment are the major income of the villagers. Given its geographical location, Horowpathana was affected by the civil war for decades (1983–2009).

In this context, the WVSL, an international Christian relief organization [23], implemented the HADP during the period 2000–2017. The main objectives of the HADP include improving education, health and nutrition, and economic development focusing on children lived in Horowpathana, a rural, vulnerable and deprived area [4]. There are four sectors of work including economic, education, health and nutrition and the sponsorship programme. A health promotion component was included under the health and nutrition sector (2012–2017). To ensure sustainability, the final period (2016–2017) included a progressive responsibility shift towards the communities [4].

### Significance and aim of the current study

More intensive research or evaluation is required to understand the relevance, effectiveness, impact and sustainability of an implementation [25]. Evidence suggests a significant research gap on implementation strategies in economically challenged countries [21, 26, 27]. According to HADP final evaluation report, the changes in the health and nutrition sector remain the strongest among all initiatives of the project [4]. As this evaluation was not independent of the WVSL, it lacks details about activities and participants expressed doubts regarding sustainability. Therefore, there is a need for complementation and it is important to explore the sustainability of the outcomes after the project’s conclusion; especially once the external support and sponsorship is over. In this study, we aim to provide a complementary evaluation, specifically focusing on the sustainability of the programme, from the perspectives of mothers who participated in the HADP.

## Methods

### Document review

A thorough documentation review was carried out to understand the project outcomes. Specific intervention content is rarely described in detail in the evaluation report. According to the HADP evaluation report, the selection, implementation and monitoring system of the overall programme are seen as a participatory mixed-methods approach [4]. To ensure sustainability, the final period (2016–2017) of the project has included a progressive responsibility shift towards the communities. Despite this and the fact that the evaluation generally showed high satisfaction, a majority of participants expressed reservations regarding sustainability. 74.2% of the participants agreed or strongly agreed on the statement “When WV leaves, the community will need to find another organization to help themselves” and still, 35.9% agreed or strongly agreed on “Once the sponsorship programme is over we could not survive.” ([4], p. 33).

Accordingly, the expected outcomes of the current study are;

1. To provide stakeholders of the HADP with a complementary short-term follow-up evaluation of the social sustainability of the programme’s outcomes from the perspectives of mothers who participated in the project.
2. To identify components suggesting sustainability of the HADP from the perspectives of mothers who participated in the project.

## Research questions

In order to address the aim and expected outcomes, the following research questions were developed.

Primary research question: How do mothers participated in the HADP describe the programme and its effects four months after the conclusion of the programme?

Sub-questions included the following.

- What benefits and behavioural changes related to the HADP do mothers describe?
- How do mothers describe community efforts and challenges to maintain the benefits? What adaptations do they describe?
- How do mothers describe social sustainability aspects of the HADP?

## Research design

A descriptive qualitative methodology was chosen to address the aim and objectives. Qualitative studies are more sensitive to contextual aspects, can provide important insights into health promotion interventions [13, 28] and complement quantitative studies [29].

**Fig. 1:**
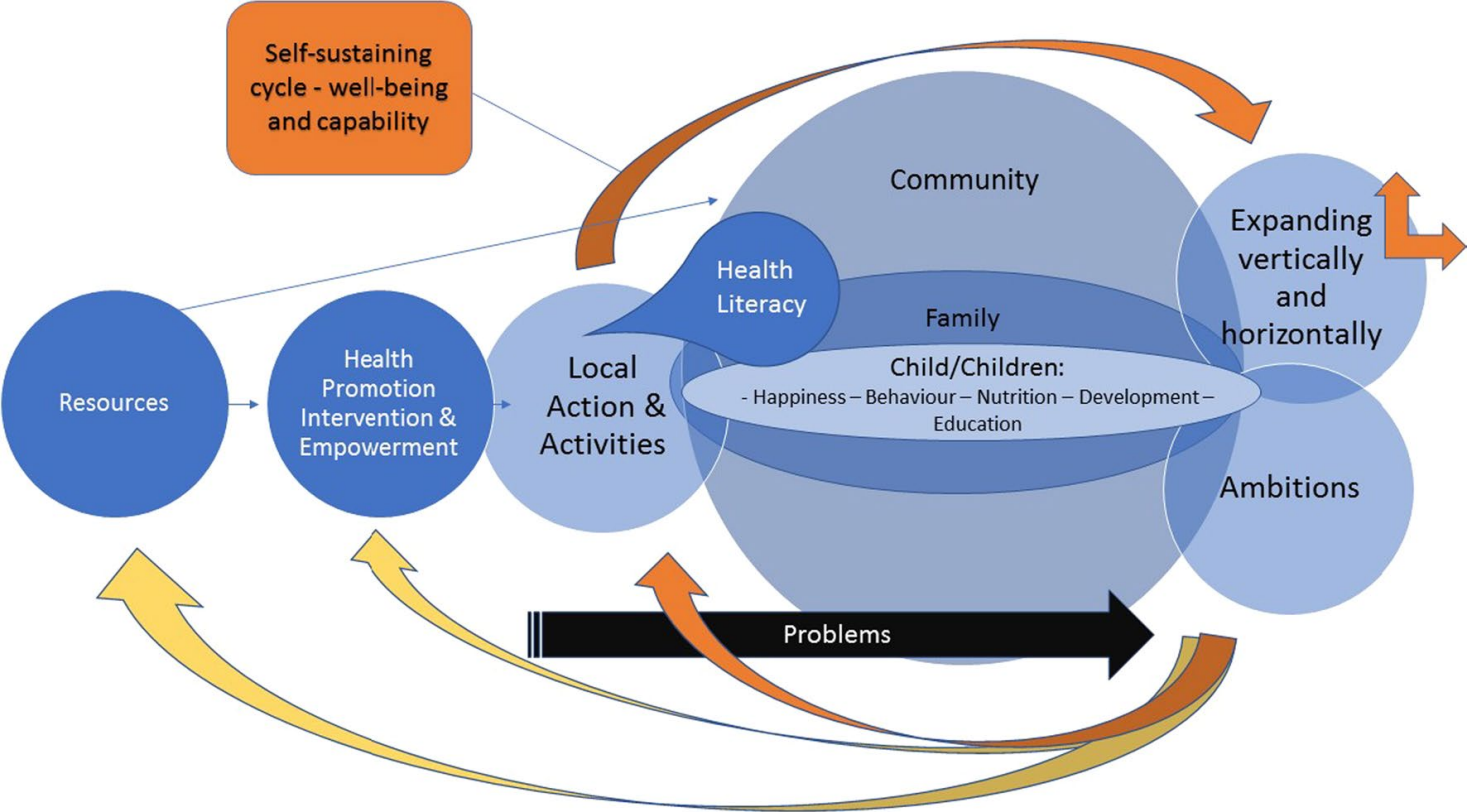
Development model of implementation for sustainability

A hypothetical model was developed (See Fig. 1) to visualize and structure the context of the study [30]. Evaluation indicators of child well-being were developed in line with UNICEF guidelines—the work of Howard White and Shagun Sabarwal [25]. A semi-structured interview guide was developed based on the above. (See Additional File 2)

### Participant recruitment, data collection and analysis

Mothers who had participated in HADP health promotion activities for at least one year and were a parent to a child of age five or less during their participation were considered eligible to take part in the study. Convenient sampling was used as the time and resources were scarce. Recruitment of participants was done through a health promotion facilitator who had worked in the HADP as the interviewers were not familiar with the programme and the study context [28, 31]. A sample of 15 mothers was identified. To answer qualitative in-depth research questions, small samples are recommended [32] and the data collection was carried out till the data saturation was achieved. Written informed consent was obtained for voluntary participation. All interviews were conducted in their native languages (either Sinhala or Tamil) over five days. Trained research assistants (RAs) moderated the interviews and translated the content to the student researcher (AA) during and after each data collection session. When necessary, AA probed questions for further clarifications [33]. Accordingly, fifteen in-depth interviews were conducted and data analysis was carried out using the content analysis method—an interpretive and naturalistic approach that is both observational and narrative in nature.

All interviews were audio-recorded, transcribed verbatim and later translated into English with the assistance of a professional translator. Preliminary data analysis was commenced in the native languages as well as in English. This helped AA to be familiar with the data set, modify the interview guide (after 8 interviews) and determine whether the saturation is achieved. Seven more interviews were conducted along with continued data analysis till saturation was achieved. Engagement with the participants helped AA to build trust and rapport, which ensures true, rich and detailed responses [34].

Obtaining good data requires interactive skills, dedication and awareness of the interview process [35]. The RAs were briefed about the study aim and the interview guide. They were trained with role-plays before the actual interviews took place. All interviews were carried out at participants’ homes, at times with other family members present. Interview duration was 40–60 min on average. Shortest interview was 12 min. As there was no new information appearing after the 14th interview, we judged the saturation achieved and data collection stopped at the 15th interview [32].

During in-depth data analysis, we compared the transcripts in the native language as well as in English in order to minimize the information lost during analysis and to make sense of findings in a more meaningful way. The data were coded manually as well as using Nvivo11. Emerging categories were constantly redefined to make them mutually exclusive and collectively exhaustive [35]. When all relevant information was categorized, we condensed it [35] and reoccurring themes were identified. Subsequently, the abstracted information was double-checked against primary themes.

The guiding tool for category abstraction was the Dahlgren and Whitehead model illustrated in Fig. 1 [9] and the working definition of sustainability [36]—*Four Months after conclusion, activities and behavioural changes of the families who participated in the HADP are maintained on community and individual level; they are adapted if needed and still considered to be beneficial by the community* (Additional file 1).

## Strengths and limitations

The short data collection time is a strength as there was little time for participants to communicate interview content. Doing interviews in a formerly colonialized country as a privileged white male European academic is likely to impact participants’ willingness to speak openly. Having trained RAs or translators during the interviews minimized the communication gap and increased the interest. Probably, the women’s answers may have differed if talking to female [28] and/or local interviewers [37]. We addressed this within our scope by choosing local female translators. The sample consisted of several mothers who were reported to be community mobilizers or key persons. This might produce an overrepresentation of positive reflections.

## Results

Fifteen mothers from Horowpathana area participated in interviews. Twelve mothers spoke Sinhala language, and the rest of the mothers spoke Tamil. Their ages ranged from 21 to 41 years. All participants had at least one child of age five or less during their participation in HADP. All of them worked primarily as housewives and caretakers of their children. Paddy cultivation (which is harvested twice a year) and chena are their main livelihood activities.

During interviews participated mothers attributed diverse perceptions on child well-being, changes in everyday life as a result of HADP and sustainability of the changes in line with the theme “health literacy”. The definition of health literacy emphasizes people’s ability to use health information rather than just understand it, ability to make “well-informed” decisions rather than “appropriate” ones, incorporates a public health perspective and acknowledges that organizations have a responsibility to address health literacy [38].

Two sub-themes emerged: transformation for betterment and sustainability. The sub-theme, “transformation for betterment”, consists of three categories: beneficial changes at individual, family and social or community levels. Sub-theme sustainability consists of two categories: (1) drivers or adaptations for continuation and (2) determinants that hinder the continuation. Table 1 summarizes the key findings.

**Table 1:** Table of sub-categories, categories and themes

Sub-categories	Categories	Sub-theme	Theme
Early Childhood Care and Development (ECCD)	Beneficial changes at the individual level	Transformation for betterment	Health literacy
Child’s nutrition and weight			
Child’s happiness			
Child’s education			
Reduction of alcohol, cigarettes, drugs usage	Beneficial changes at the family level		
Day-to-day family life and economy			
Mothers’ knowledge about parenting			
Family happiness			
Collective activities—Playhouses, Baby rooms, community activities	Beneficial changes at the community/village or society level		
Social connectedness—Children’s societies, Mother supportive groups			
Empowerment—carrying out the activities alone, spreading it among new mothers and other villages	Drivers and adaptations for continuation	Sustainability	
Future plans- Individual plans, collective goals			
Suggestions for improvement			
Personal issues of mothers—financial issues, time, lack of motivation	Determinants that hinder the continuation		
Group behaviour—Interpersonal issues, conflict of interests among group members			
Contextual issues and barriers—social pressure, sabotaging			

### Sub-theme 1: transformation for betterment

This sub-theme describes the mothers’ perceptions on beneficial changes that occurred in the project area, at the individual, family and community levels as a result of HADP. According to mothers, the children of the Horowpathana area showed beneficial changes in their early childhood development, eating patterns, weight and nutrition, overall happiness and school performances.

#### Early childhood development

Concerning ECD, some of the mothers referred to motoric advancements (rolling over, crawling, walking) and cognitive iterations (talking, identifying objects and colours) of their children being completed at early ages.

“There is a big development of our babies when compared with same-age children in other villages. Almost all mothers in our village did [ECCD activities introduced by the HADP]. My baby sings songs alone, identifies one or two colours alone, and when playing, he is more active than others” (S 7)

“We compared CHDRs of our children during the programme with CHDRs of same-aged children before the programme. Our midwife miss helped us to do this. Before this programme, a 12-month-old baby has spoken about 10 words, an 18-month-old baby has spoken about 20 words. Babies who took part in the program could speak about 50 and 75 words at similar ages.” (S 3)

#### Nutrition and weight of the children

The mothers reported how their eating patterns changed as a result of HADP discussions.

“All the mothers in the village started keeping records of children’s food intake in a ‘nutrition book’. We scheduled meal times and provided them with various nutritious meals. We learned creative ways to prepare healthy and balanced meals from low-cost food items available in the village. We introduced a reward system for children. As a result, their weight is gained. Children became healthier.” (S 1)

“Mine was an underweight baby before I took part in this intervention. If you look at his [CHDR] you can see how his weight has gained during the programme.” (S 2)

“Hapana calendar” is a tool used by mothers to measure a child’s daily food intake, eating patterns with a reward system. Children received a star based on how much they ate and what they ate. Collective feeding at playhouses that were set up during the programme had become a regular event in their lives. Mothers shared the food items they cooked so that all their children received a nutritious meal. With play, eating became a pleasurable activity for children.

“We fed our children what we grew in our garden. Fruits and vegetables… We did not buy expensive stuff. We displayed ‘Hapana-Calendar’ at playhouses so the child could see. If the child ate a little for lunch, we gave a small star and if he ate all we gave him a big star. It changed the amounts, food preferences and eating pattern of my baby.” (S 2)

Another beneficial change that occurred due to HADP is lowering the consumption of sugar, oil, salt, chilli and rice while eating more vegetables, dairy, eggs and fish. Besides, the consumption of junk food, biscuits, sweets, crisps, soft drinks and artificial flavours (which they referred to as “foolish food”) was reported to have declined. This beneficial change was not only in children’s attitude and behaviour but the entire family.

“Children refused to buy such snacks. They now request home-made sweets instead of biscuits or toffees” (S 3)

#### Happiness

HADP resulted in increasing their children’s happiness. The “Happiness calendar” is a simple, subjective tool used in many health promotion interventions to assess the emotional well-being of individuals in terms of feeling happy, sad or angry on a daily basis. People use colours and emojis of faces to represent their respective emotions (i.e. anger, worry, happiness).

“During the programme, we started marking the happiness calendar. Children did it for the entire family. It became a habit that we discuss our daily mood. For example, why the child is sad and who made him sad. Because of the calendar marking, overall family happiness and unity increased.” (S 4)

Children were involved in marking this home-made calendar and evaluated how individual moods influence family happiness. Marking happiness calendar was reported to be useful in enhancing family connectedness, reducing family disputes and alcohol or drug usage among fathers there by increased care and affection towards children.

#### Education

Several mothers commented that their children’s educational performances were improved as a result of HADP. Early school dropouts and drug or alcohol usage among teens had been a common issue in Horowpathana. However, during HADP there had been a noticeable reduction. A mother said:

“Children who took part in the programme were lucky to realise the importance of education. We saw an increased interest of children for studies. Their school performances got better.”

HADP resource persons had discussed effective studying techniques and the importance of teaching children collectively. Through mother supportive groups they started the concept of community-based “evening schools” at the village level, usually at the playhouse. Some parents, grandparents and/or older siblings were involved in teaching younger children their subject matters and even life skills.

“Through this, our children became more enthusiastic towards learning. Not only from the school teachers, but they learned many things in other ways. Their motivation was high. I was pregnant when I joined this program. When compared to my elder son, I have high hopes on the youngest as she was much benefitted through this program.” (S 4)

#### Reduction of alcohol, cigarettes and illegal drug use

This is another noteworthy change at the community level. According to participants, HADP interventions resulted in reducing alcohol or drugs and smoking consumption among village men, thereby fewer family disputes. The HADP raised their awareness on how much money they can save if abstained from alcohol, illegal drugs and smoking. Even after the conclusion of the project, they maintain “expenditure books” in which they keep records of their expenditure and savings at the family level as well as village level. Village women were able to make aware their husbands through displaying motivational posters used for campaigns at home and using empty beer cans as flower pots or garden decorations. As reported by participants, most of the men either reduced or stopped their consumption of alcohol or cigarettes after confrontation, becoming aware of amounts of alcohol intake, related expenditures and its impact on their family life.

“I marked [in the expenditure book] the amount of beer, liquor, cigarette which my husband bought. When I showed this to my husband, he realized that we have been spending a lot of money on useless things. So, he reduced those expenses” (S 8)

“Before the programme, thirteen shops in our village sold cigarettes. But after our campaign, none of them sells cigarettes now.” (S 5)

#### Everyday life and family economy

The HADP introduced several monitoring tools to the villagers so that they could measure changes in their day-to-day life. For example, the happiness calendar stimulated conversations about well-being and happiness among family members. The expenditure books helped distinction of “necessary” and “unnecessary” expenditures; cutting down unnecessary expenses: men educing alcohol or smoking-related expenses, fewer expenditures for unhealthy food; families were economically better off and improved their quality of life. The finances became more transparent. Some mothers reported using recycled or natural material for toys rather than buying new ones.

“Expenditure book! Before making the expenditure book, I didn’t know how much we earned and spent. After making it, I knew, and I could save money. It benefited the whole family.” (S 7)

Home gardening was another beneficial change for families.

“We had fresh food to eat. I used the saved money for children’s private classes”. (S 1)

One mother reported that her husband started fulfilling the expected father role better after taking part in a discussion, by helping children with their homework.

Furthermore, mothers reported that children and other family members reduced the TV time and increased more together time. Children show good attitudes and behaviour changes, for example, reduced use of mobile phones, caring for elderly and helping parents in household chores.

#### Mothers’ knowledge on parenting

The mothers expressed that their knowledge and skills about parenting improved through the HADP. They brought posters that displayed diagrams and charts to show their knowledge gain. Topics that brought up were: feeding techniques, nutrition of infants, identification of underweight or overweight, vaccination ages, home-based activities to promote early childhood development, sensory stimulation, nutrition and care during pregnancy, pedagogics, connections between alcohol consumption, family well-being, education and play, overall health concerns and prevention of non-communicable diseases.

“Before I learned these, I hated feeding time as the children refused eating what I cooked. With collective feeding they ate happily while playing with friends.” (S 6)

“I was pregnant when I joined the programme. I made a nutrition book for me, marked the support I received from my husband and family and created a baby room for my daughter. When she was born I knew how to and I was well prepared to feed her five senses!” (S 4)

Another noteworthy change of mothers as a result of the HADP was they started using several measurement methods to monitor changes and the overall well-being of their children. For example, comparison to other children, Child Health Development Record (CHDR) [39], grading school performances, documentation of weight and related activities in CHDR or nutrition book, happiness calendar and comparison of child’s situation to standard values such as birth weight, BMI. There is enough evidence to show how mothers were enabled to use their understandings to take better, informed decisions through measuring changes using health-related indicators. As described by them, their confidence increased over time as they were more engaged in these activities which now has become a daily routine in their lives.

#### Overall changes at the community or village level

As reported by mothers, there are several positive changes seen in the communities that took part in the project. Early school leaving of teens, child marriages and teenage pregnancy rates had been high but declined during the period covered by HADP and after. Child abuse was reported to be high in some villages, but the mothers highlighted how a feeling of general responsibility for children was created among adults in those villages as they were involved in developing playhouses, baby rooms and engaged in ECCD activities. Further, money management; improving family economy; reduction of alcohol, illegal drugs, smoking consumption; and increase in collective social activities, caring for elderly and increased unity, reduced selfishness and discrimination among people are noteworthy changes that continues even after the conclusion of the project.

“Our villages used to be cornered and unnoticed. But the project helped us create a safe and better place for our children. We have hope for our children’s future now.” (S 5)

The project has brought several noteworthy beneficial changes to society. There are improvements seen in social structure and connectedness in communities through involvement and engagement of collective, communal activities. After discussions on sensory stimulation and ECCD, the mothers helped each other to create baby rooms and communal play areas for children in the village. Such playhouses that were built during the programme still serve as common meeting places for children as well as parents. On our visits to some of the baby rooms and playhouses, we saw availability of educational, play and reading materials (which were age-specific and ECCD focused), and the regular monitoring tools such as sensory and motoric development charts, activity records maintained by parents.

“Playhouse gatherings were very attractive. Fathers build the play items. When all were together, mothers noticed underweight children, gave them nutritious meals collectively. We all played and sang together with children.” (S 15)

#### Children’s societies and mother supportive groups

Social connectedness among children has enhanced through children societies, while mothers were connected through their groups. This helped children to feel responsible and empowered as their voices were heard and they took leadership as change agents.

“Children society gatherings were once a month. Mothers guided the children. Through their societies, children took the leadership to address matters like reducing alcohol consumption, cleaning irrigation tanks, child safety and dengue prevention. They conducted a poster campaign and a health camp.” (S 11)

The HADP has resulted in several mother supportive groups in all villages in Horowpathana area. Every group has at least one community mobilizer who took leadership in many health promotion initiatives. Their activities included fundraising through communal activities like fairs, carrying out activities to promote ECCD, awareness of child protection, support children in their education; interventions to minimize low birth weight, teenage pregnancies and child exploitation at village level and also support families to overcome poverty through home gardening or self-employment. Mother supportive groups take leadership and facilitate coordination of health promotion activities in all villages. They are collectively involved in building playhouses and maintaining those; support in establishing baby rooms; excursions with children; community actions such as cleaning the environment; and attending to elderly people who need support and organizing children’s day concerts.

### Sub-theme 2: sustainability

This sub-theme describes the promoters and hinders for continuing the beneficial changes they experienced after the conclusion of HADP project. The findings prove that even after four months, majority of the mothers had similar interest and enthusiasm to carry out the good work they initiated through the HADP.

“Even though the World vision programme has stopped we are still practising the process they taught us. Now we discuss those things in mother supportive groups so that new mothers also practice those things.” (S 8)

#### Drivers and adaptions for continuation

All interviewed mothers were seemed to be empowered through the HADP, and they have individual and collective goals to be achieved and suggestions to improve current health promotion practices.

#### Empowerment

“Through this project, we learned many things. [The project team] created self-confidence within us. They taught us how to speak without fear. Actually, I can talk with elite people and tell them ‘We want this!’. I am that confident.” (S 6)

Many mothers play a role as volunteer community mobilizers, and they continue to spread the process and share their knowledge among other people in their village or other villages. More new mothers have started certain activities after observing these “role models”.

“We have done programs at other places. We go to these places. Show them what we did, how we did and what we gained out of these. The way I see it, we are confident, independent women.” (S 14)

After the project, mothers continued to work out creative and novice actions to improve the health and well-being of their children, family and entire village using the concepts they learned through the project. Activities that improved weight and nutrition of children (nutrition book, nutritious food for underweight children, collective feeding practices); activities to reduce overweight; religious or spiritual activities with children; excursions; (re-)building playhouses collectively with weatherproof material; collective gardening; maintaining expenditure books; and organizing children’s birthdays or new year events were in their list of continuing activities.

They had individual as well as collective plans for further improvement. Several mothers were planning to pass on their positions held in their mother supportive groups to newcomers so they will also take responsibility.

“We started this group in 2015. Since then up to now, I was the secretary. We have a plan to give the chance to another mother and I would give my maximum support to her” (S 4)

#### Suggestions for improvement

Many mothers expressed their wish for continuation to increase their knowledge further and also to raise awareness of others who did not participate in HADP. Among the suggestions, mothers brought various ideas regarding popularizing and the dissemination of good practices among others. Using social media, leaflets, newspapers and television to share their success stories; perform street theatre; and allure more were some of the suggestions. One mother suggested to continue the programme or facilitation by resource persons with shorter intervention time at nearby villages where people had fewer financial resources, suffered from diseases, and required sanitary facilities and children were at risk for sexual exploitation and violence.

“We will be able to mediate as change agents. If [resource persons] initially could help us to set the background, we can often visit those villages and continue the process. If we could do it, they can too.” (S 10)

Mothers expressed their need for educational support and material. Mothers suggested having an “umbrella organization” of all mother supportive groups. Some mothers suggested regular audits to “monitor progress of the work carried out by mother supportive groups”. The creation of a paid job as a health or nutrition officer to facilitate health promotion work at the village level was another idea.

“If we have a designated post, it will be easier for us to go to other places to teach and train other people also. It will help in the development of all areas to be illness free.” (S 1)

“The government could appoint young graduates as nutrition officers. Then they can guide us. Basic health and nutrition of the people can be improved by empowering people and increasing their health knowledge.” (S 13)

#### Issues and challenges to successful implementation

Despite the factors that facilitated sustainability, mothers also discussed the issues and challenges that hinder their enthusiasm and willingness to continue their work. Having dealt with financial hardships, most mothers wanted a paid job rather than voluntary work. Several participants pointed out their economic needs and insufficient money.

“We are working with the [World Vision] for a long time. So, if they or government give us employment, we will be fine. (…) We were able to help many children [and families] in our village. So, if we abandon this now, it will affect the future of our village and future generations too.” (S 7)

According to the mothers, not all villagers are happy about the healthy changes of people. They often get discouraged when they have to deal with negative attitudes and sabotaging activities of certain villagers, for example, alcohol vendors, drug dealers and uncooperative youth.

“Some mothers were sceptical about the programme and refrained from participation. They create problems as they would not have seen a need for change or would not have taken the programme seriously.” (S 2)

“I feel really sorry for those mothers who blamed us, even World vision. They didn’t practice 5 senses stimulations. They shouted at us for talking about biscuit consumption of their children and pulled the legs of mothers who participated.” (S 8)

In certain situations, there had been interpersonal issues between mothers that lead to conflicts and as a result, some mothers refrained from joining the activities. This caused additional stress for mothers and reduced their willingness to continue. When group members were leaving, it would often be a lot of pressure and responsibility is on the remaining members.

“Some mothers bring their children to playhouse but do not prepare meals for collective feeding. Rest of the mothers did not like it” (S 13)

“When planning a collective cleaning activity, the mother supportive group imposed a fee on non-participation. It negatively affected the unity of the group.” (S 7)

Another challenge reported by mothers is the low participation of villagers, especially fathers and teenage children after the HADP concluded.

“Perhaps when growing up, they become less interactive. It seems their interest is gradually declining” (S 1)

Another challenge mentioned was coordination between key persons and compatibility of that role with work duties. The arrangement of key persons was also described as challenging. One informant described how she was taking the key person role very seriously looking out for children. This would sometimes create conflicts with other villagers.

In addition to these, some of the mothers described challenges concerning their context. For example, in the Tamil-speaking area, there are no public spaces, and only one school had purified water and hygienic sanitation facilities. Droughts and economic hardships change the focus of people, so they become less interested over time. Some mothers preferred collective gardening, but due to limited resources, it remained in individual households. The maintenance of evening schools and playhouses was challenging with lacked support from fathers. Mothers reported that some playhouses were already destroyed by heavy rainfalls and no reconstruction had been done.

## Discussion

This paper presented findings of a descriptive, qualitative study on mothers’ perception of child well-being, changes in everyday life and sustainability of a health promotion intervention which was carried out during 2012–2017 in Horowpathana area where the last two years of the project included a progressive responsibility shift towards the communities. The current study is a qualitative evaluation and a form of triangulation for the donor evaluation report of the HADP. The findings of this study affirm the process of enabling people to increase control over the determinants that govern their lives and thereby improve their conditions [10, 11]. Participants were able to observe and measure many beneficial changes in their lives, households and entire community. Their ability to obtain, process, understand and use health-related information has improved through the HADP [38].

The key theme “health literacy” described the participants’ ability to acquire new knowledge relating to health and use that knowledge to make informed decisions to improve the existing health and well-being of the communities and their members. Findings show the healthy changes occurred on individuals, families and entire villages as a result and their efforts towards the continuation of good practices even after the conclusion of the HADP. Improved health literacy enabled the mothers to positively influence several determinants of their children’s health and empowerment resulted in sustainability [9, 25, 36].

As per the findings, components of sustainability are healthy changes in diets, sounder expenditure patterns and a child-friendly environment. The holistic manner how mothers spoke about well-being and connections between benefits can be understood as suggesting social sustainability, as this indicates a health promotional way of thinking. For example, many of the changes on the individual level were linked to changes on the family or community level, which is in line with key principles of health promotion [10] and the Dahlgren and Whitehead model [9]. When asked about continuation, many mothers mentioned improved knowledge as the most significant component which is an important aspect of health literacy.

Findings affirm that the outcomes of HADP are effective and sustainable. Community-owned collective actions sustain by self-monitoring mechanisms. Shared enthusiasm and knowledge of the community members are the key to success [40, 41]. Evidence from other Sri Lankan settings affirms the effectiveness of community-based health promotion programs in improving ECCD practices, child protection, nutrition, education, social services, underweight and low birth weight, family happiness and well-being, prevention of smoking and alcohol, NCD prevention among lay communities, especially with those who have less formal education [40, 42–44].

Health promotion interventions help the lay communities especially those who are living in the most vulnerable and disadvantaged areas as its focus is empowering people. Findings show that the mothers were empowered through the HADP to carry out actions to improve child well-being as the mothers purposefully stimulated five senses and documented children’s development, which is another example of obtaining and processing health-related information, thus showing improved health literacy. Anyhow, the end of the civil war (2009) and general development may contribute to improvements in Horowpathana’s overall situation and are difficult to distinguish from programme outcomes [18, 29]. However, improving health literacy has contributed positively in people’s compliance in improving community-level preventive health sector activities. For example, people have taken leadership in reducing the risk of non-communicable diseases, dengue prevention and prevention of alcohol and cigarette consumption among village men.

Findings show behavioural and structural changes in the everyday life of families and community members were beneficial for the entire community. Outstanding was the happiness calendar as a practical tool [43] that improved the mothers’ health literacy by making patterns transparent and measurable. The higher cohesion between mothers and their partners, not least due to the reduction of alcohol or drug consumption of men, was necessary as a social foundation for sustainability on the family level [42]. The mothers were motivated to carry on the activities and emphasized that a socially sustained change in attitudes had happened. For example, fathers were measuring up the mothers’ expectations, mothers continued to monitor and document changes and health-related factors even after the conclusion of the HADP, which shows increased awareness and health literacy in families. On a community level, mother supportive groups and children societies facilitated discussion forums for health-related topics and catalysts of needs. These findings reaffirm the empowerment and sustainability in line with the health promotion approach.

Despite the success stories, the findings show certain red flags that hinder the sustainability of the beneficial changes. Among the factors that negatively affect sustainability, the continuation of the mother supportive groups was in an uncertain state as the sense of responsibility and level of enthusiasm varied over time, especially, when the workload increased and have to deal with other hardships of day-to-day life. Studies from other settings also highlight that social determinants such as sabotaging and demotivation by others are common challenges in community work [45]. It is important to explore the concerns and motives of non-participants to understand such determinants. The donor evaluation report suggests using a human resource management plan and monitoring from both WVSL and the community [4]. The described challenges were addressed with schedules, work share and fees for non-participation in some villages, but these did not always bring full and equal participation. As suggested by the participants, a systematic pathway to monitor and evaluate the progress (for example, audits, follow-ups or having an outsider as a field officer) will improve the chances of sustainability of these health promotion interventions.

In villages where the people show more unity and connectedness, the mother supportive groups had become well-functioning organizations. This goes along with the principle of health promotion—strengthening social networks [14]. Nevertheless, it is important to investigate the long-term endurance, adaptations and evolving of those groups. Adaptation can refer to either change in the program or implementation strategies or change in an individual’s maintenance of behaviour [36]. It seems important to note the inter-relatedness of above key concepts. Adaptation or evolvement needs to continue to produce benefits to be sustainable eventually. We point out one mother’s suggestion: to create paid jobs for key persons. This supports the theory that empowerment through knowledge can create professional health promoters [11] to whom community members turn. Lay people can be trained in medical topics, by increasing their health literacy [44]. One way to address this could be to acknowledge and reimburse the work of key persons. However, it is important in the implementation of health promotion programmes to consider power structures in the villages, who is chosen as key persons and to discuss balanced responsibility [12, 14, 18, 46].

Both individual baby rooms and public playhouses are beneficial outstanding outcomes. However, it is somewhat ambiguous that usage of natural material for the playhouses is environmentally and economically sustainable but was not adequate for weatherproof construction.

Findings affirm that the poor infrastructure and junction of some communities were a challenge, which can influence the cooperation with communities [21, 47]. The experience of empowerment through higher health literacy resulted in participants speaking up on their behalf and approaching authorities with self-confidence [48]. Political initiatives have formed, demanding better health infrastructure. Community building has been described to support communities in problem-solving and addressing political issues [29, 49] and to raise awareness of broader social and political interrelations [12, 50]. Those theories and findings can be confirmed by our study.

## Conclusions

Health, empowerment and related improvements in children’s well-being are benefits pointed out by the mothers as being sustained on the individual, family and community levels, integrated into everyday life with high motivation to continue. The mothers’ perspectives on health literacy emphasized people’s ability to use health information to make “well-informed” decisions that incorporate a public health perspective. They also acknowledged the responsibility of social organizations to address health literacy. From a health promotion perspective, the findings of our study indicate that people and organizations can use their health literacy skills to improve the health and well-being of their communities and its members. The sustainability of health promotion interventions depends on the level of empowerment, motivation and commitment of individuals and communities and follow-up monitoring. Some groups remained active and well-functioning, while others expressed signs of exhaustion of community mobilizers, decided not to engage longer or had grown away from other villagers. This can be a challenge to sustainability and raises questions regarding possible side-effects of empowerment and balances of bottom-up and top-down approaches, particularly considering that only 4 months passed since closure. We recommend investigating those themes further as they may result in the discontinuation of mother supportive groups.

It is possible that formal institutionalization may not suit the mothers’ needs. The adaption of the organizational form can also be interpreted as a form of ownership. Empowerment in the form of higher health literacy and consequent demands regarding structural and political issues is a sustainable success of the HADP. Higher health literacy leads to individual empowerment, community organization and development of broader ambitions towards community health and well-being. However, challenges in the form of unequal participation and high workloads of key persons require modifications of intervention and process.

## Supplementary Information

**Additional file 1.** Development of the hypothetical model.

**Additional file 2.** Interview guide.

## Data Availability

The datasets used and/or analysed during the current study are available from the corresponding author on reasonable request.

## Abbreviations

BMI: Body mass index
CHDR: Child health development record
ECD: Early childhood development
HADP: Horowpathana area development programme
RUSL: Rajarata University of Sri Lanka
WHO: World Health Organization
WVSL: World Vision Sri Lanka
