# Supplementary material for: Perceptions of mothers on child well-being, changes in everyday life and social sustainability: lessons learned from a community-based health promotion programme in Anuradhapura District, Sri Lanka
Source: J Health Popul Nutr. 2022 May 13;41:20. doi: 10.1186/s41043-022-00295-w (PMC9102598; doi:10.1186/s41043-022-00295-w)
Supplement: Supplementary file 1 — Additional file 1. Development of the hypothetical model. [file 41043_2022_295_MOESM1_ESM.docx]

**Additional file 01**

**Development of the hypothetical model**
With our understanding of the Horowpothana Area Development Programme, the model was accordingly adapted.
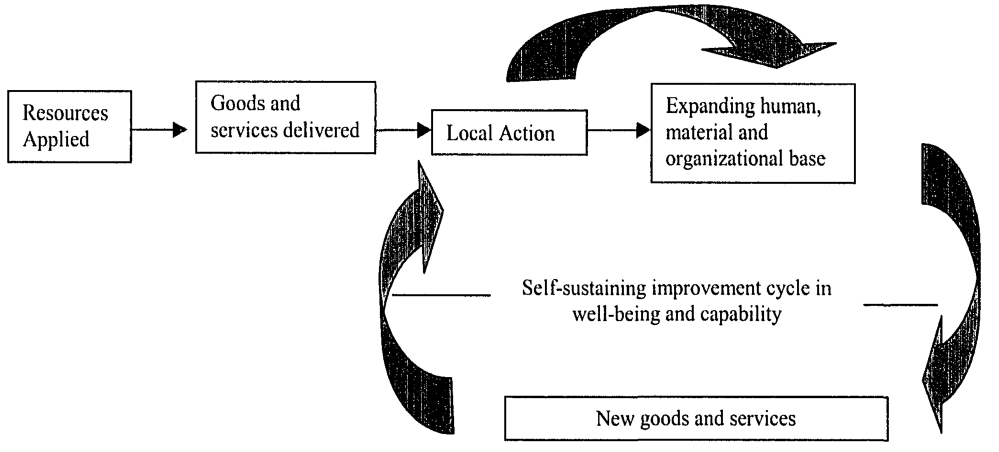

Figure: A development model of Implementation for Sustainability, Honalde & VanSant (1985) in [34]

In the first Version, we basically changed the terms according to the Health promotion intervention and recent literature (e.g. “Goods and Services delivered” became “Health promotion intervention & Empowerment”; “New goods and services” became “New objectives, initiatives, ambitions”) and we put the child with its family and community in the centre of the “self-sustaining improvement cycle in well-being and capability” in order to represent the central role they have in Health promotion.

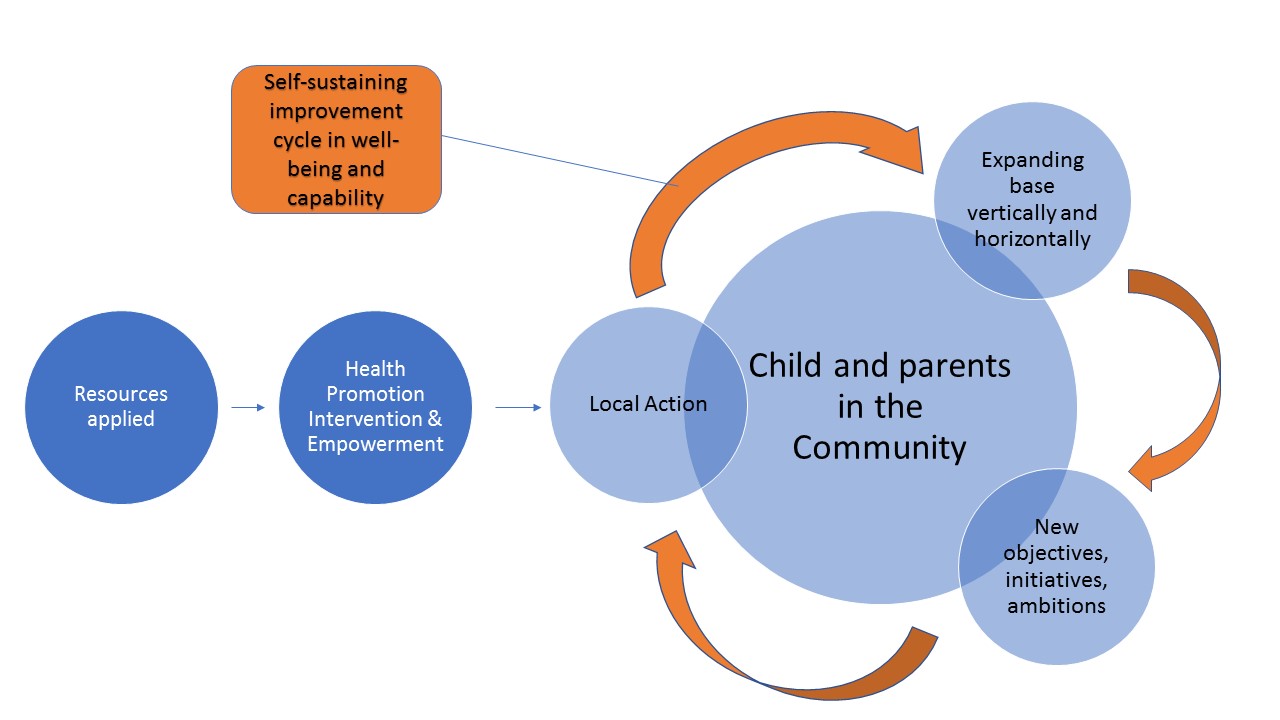

Figure: A development model of Implementation for Sustainability, Version 1, adapted from Honalde & VanSant (1985) in [34]

The second Version includes the different concepts related to child well-being, distinguishes the family and child from the community as we wanted to describe the benefits and changes on different levels and the representation of “Health promotion intervention & Empowerment” was moved closer to “Local Action”. This is supposed to represent that those are often intertwined due to the community-based manner of the intervention and that they interact closely.

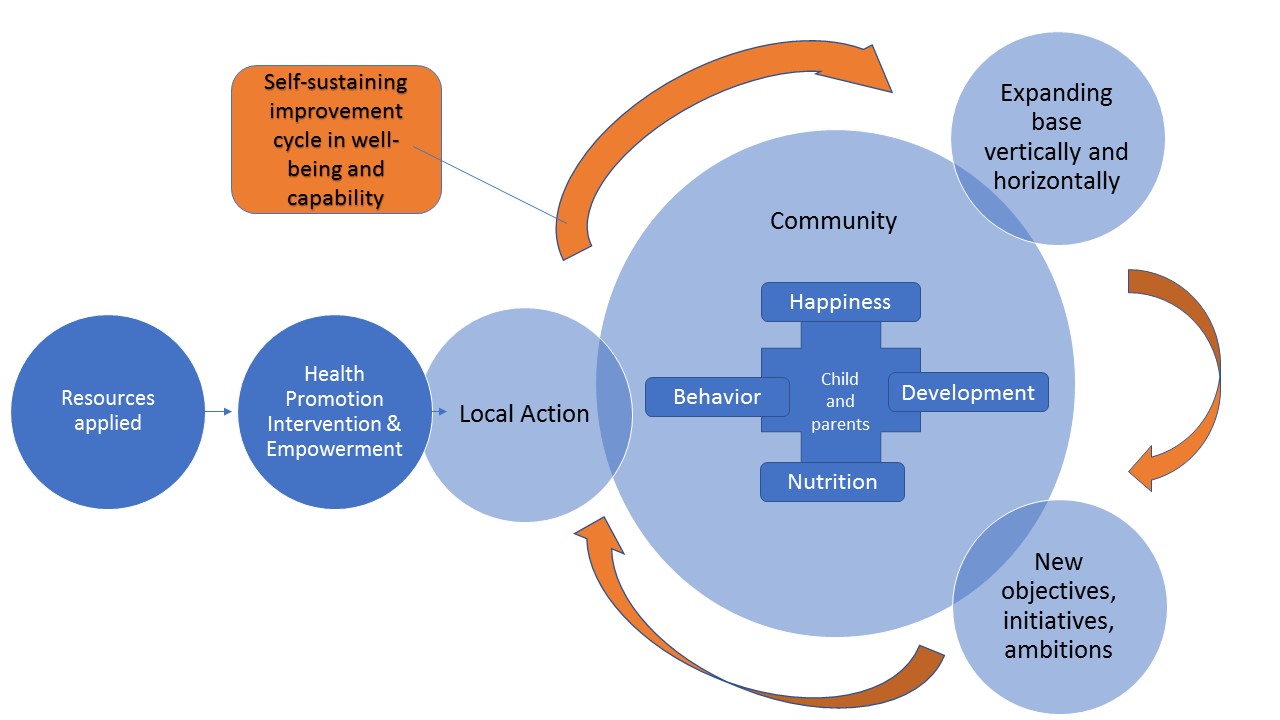


Figure: A development model of Implementation for Sustainability, Version 2, adapted from Honalde & VanSant (1985) in [34]

In the final Version, that represents our understanding of sustainability in the HADP after analysis of the interviews, new pathways are important. The darker arrows represent the ideal case, the “self-sustaining improvement cycle in well-being and capability”. From our understanding, the points of “Expanding base…” and “Ambitions” go closely together and are not – as in the prior version – consecutive. Some communities might e.g. even have new ambitions without expanding their base and vice-versa. The expansion or dissemination of the programme both to other communities and towards e.g. authorities or other societal classes is one intention of Health promotion and therefor also displayed in darker colour, i.e. part of the self-sustaining cycle. The lighter arrows show ambitions that relate back to the intervention or to Resources that are needed in the community but do not necessarily lead to further local action. An example might be the need for more financial support. We expanded the categories “Children” and “Family” to represent that they are connected to the cycle not only through the community but also as individual actors. The category “Health Literacy” was added as this was understood as one major factor in the mechanisms of Health promotion. The fine arrow reaching from “Resources” to “Community” represents the delivery of goods that were directly given to the community without embedding them into the intervention, i.e. with no or little conceptualization. The thick black arrow “Problems” goes against the direction of the “self-sustaining cycle”. It could be placed anywhere as it relates to all other categories but this way it represents how it works against the desired sustainability.
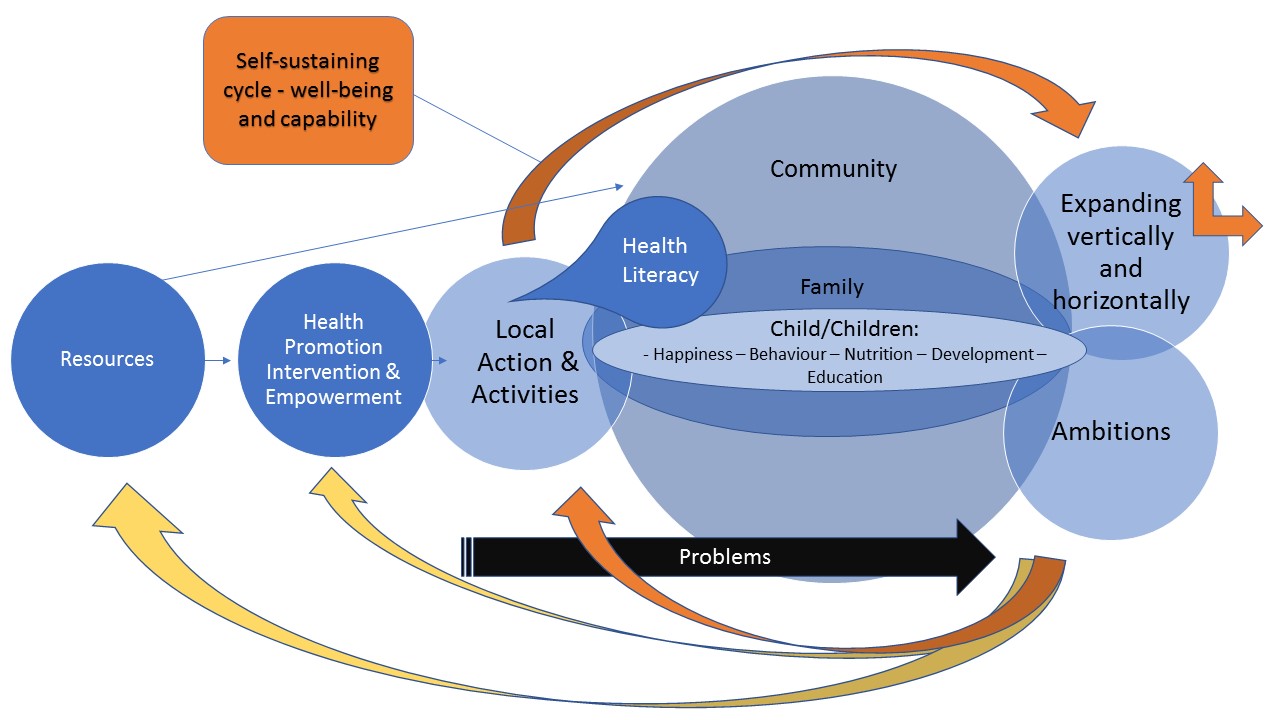

*Figure: A development model of Implementation for Sustainability, Version 3, adapted from Honalde & VanSant (1985) in* [34]
