# Supplementary material for: Perceptions of mothers on child well-being, changes in everyday life and social sustainability: lessons learned from a community-based health promotion programme in Anuradhapura District, Sri Lanka
Source: J Health Popul Nutr. 2022 May 13;41:20. doi: 10.1186/s41043-022-00295-w (PMC9102598; doi:10.1186/s41043-022-00295-w)
Supplement: Supplementary file 2 — Additional file 2. Interview guide. [file 41043_2022_295_MOESM2_ESM.docx]

**Additional file 02**

**Interview guide**

What part of the programme did you participate in?

How old is NAME?

How long have you and NAME been a part of the programme?

Tell me your story in the programme, please. *(How did you get in contact, what happened afterwards, how did it go on, what were important moments, how was the process*, *Did NAME have any changes in his/her weight?)*What are the changes in your life as a family?

What are the changes of the children
- in relation to nutrition?
- in relation to development?
- in relation to happiness?
- in relation to behaviour?

How did you measure it?

How do you think the programme’s actions will help NAME in the future?

Do you maintain what you described before (now that the programme has ended)?
🡪 How?
🡪 Are there problems in doing so?

What does the community do to maintain the previously described achievements?
*(Is this organized? Do you use any special tools to do so?)*

**How smart would you say NAME is? 1-5
How healthy would you say NAME is? 1-5
How happy would you say NAME is?1-5**

Do you have suggestions how to improve the programme?

🡪 The quantitative questions (in bold) were supposed to evaluate the respective child’s well-being on a 5-point Likert-scale. In the first three interviews, the interviewer spontaneously refrained from asking the mothers to evaluate their child’s health, happiness and cognitive ability in presence of their children in doubt of harmful effects for the children in case of low rating. Consequently, the quantitative questions were not asked throughout the following interviews.
